# Broadband unidirectional behavior of electromagnetic waves based on transformation optics

**DOI:** 10.1038/srep40941

**Published:** 2017-01-20

**Authors:** XiaoFei Zang, YiMing Zhu, XueBin Ji, Lin Chen, Qing Hu, SongLin Zhuang

**Affiliations:** 1 Shanghai Key Lab of Modern Optical System and Engineering Research Center of Optical Instrument and System, Ministry of Education, University of Shanghai for Science and Technology, No. 516 JunGong Road, Shanghai 200093, P.R. China; 2 Terahertz Science Cooperative Innovation Center, Chengdu 610054, P.R. China; 3 Department of Electrical Engineering and Computer Science and Research Laboratory of Electronics, Massachusetts Institute of Technology, 77 Massachusetts Avenue, Cambridge, Massachusetts 02139, USA

## Abstract

High directive antennas are fundamental elements for microwave communication and information processing. Here, inspired by the method of transformation optics, we propose and demonstrate a transformation medium to control the transmission path of a point source, resulting in the unidirectional behavior of electromagnetic waves (directional emitter) without any reflectors. The network of inductor-capacitor transmission lines is designed to experimentally realize the transformation medium. Furthermore, the designed device can work in a broadband frequency range. The unidirectional-manner-based device demonstrated in this work will be an important step forward in developing a new type of directive antennas.

High-directive antennas, as functional elements to concentrate radiation in a desired direction, have been successfully applied in various systems *i.e.,* radar, satellite communication, microwave power transmitting systems, and so on^1^,^2^,^3^. Traditionally, there are three kinds of high-directive antennas, namely reflectors^4^,^5^, array of radiating elements^6^ or cavities^7^,^8^,^9^ and lens^10^,^11^,^12^,^13^ (such as Luneburg lens antennas and half Maxwell fish-eye lens antennas), which can be realized by using reflectors, a set of individual antennas, Fabry–Perot resonators, and gradient-refractive-index (GRIN) metamaterials. Furthermore, transformation optics (TO), as a useful tool to control electromagnetic waves, provides a systematic method to manipulate wave propagation using novel wave-matter interactions, resulting in many new applications such as invisible cloaks, field rotators, beam splitters, electromagnetic black-holes, super-scatterers, tunable electromagnetic gateways, *etc.*^14^,^15^,^16^,^17^,^18^,^19^,^20^,^21^,^22^,^23^,^24^. In essence, TO is an attractive concept to manipulate the field distribution of electromagnetic waves. Therefore, as a new design approach, TO was also extended to design high-directive antennas in recent years. And, many kinds of high-directive antennas such as reflector-dependent high-directive antennas^25^,^26^,^27^, multi-beam lens antennas^28^,^29^,^30^,^31^, super-antennas^32^, and Luneburg lens antennas^33^,^34^,^35^ were proposed and designed based on TO. Although all of them are commonly characterized by high-directivity and broadband response in theory, the corresponding materials always require extreme values (anisotropic and inhomogeneous transformation medium), and most of them are dependent on the reflectors. In addition, for the TO-based flat Luneburg lens, most of the input energy leaks into the free space rather than transferring into the directional emission (In fact, the traditional Luneburg lens antennas and half Maxwell fish-eye lens antennas are also subject to this defect). In addition, conformal-based unidirectional and high-directive antennas^36^,^37^ were also proposed (with the inhomogeneous and isotropic transformation medium), but they were still dependent on the reflectors too. Recently, dielectric singularity was proposed to act as reflector^38^. When a line current located in a fixed position and coated with isotropic but inhomogeneous transformation medium, the electromagnetic waves (generated by the line current source) can be confined propagating in one specific direction without the need of reflectors, resulting in the unidirectional behavior of light, in theory. However, it is still hard to be realized in experiment due to the inhomogeneous transformation medium (It means that we need to design multiple layers of GRIN metamaterial structure to realize the inhomogeneous transformation medium). In addition, the corresponding excitation source can be just located in a fixed position due to the dielectric singularities. In a word, most of the previous works related to the TO-based directive antennas are limited to theoretical analysis and numerical simulations (with complicated material parameters), lacking the corresponding experimental demonstration.

Here, in this paper, anisotropic but homogeneous transformation medium is proposed and implemented to obtain the unidirectional emission of electromagnetic waves without the use of reflectors. Such a transformation medium is fabricated by controlling parameters of inductor-capacitor (L-C) unit cells in a transmission-line network. Our simulated and measured results demonstrate that such a novel device can work over a broad spectral range (about from 15 MHz to 75 MHz). It should be noted that the unidirectional effects proposed in this paper are realized by three kinds of transformation medium, so we just need to design three kinds of L-C unit cells to simulate transformation medium, and fortunately, the position of excitation source is not fixed but flexible.

## Theoretical Model

**Figure 1:**
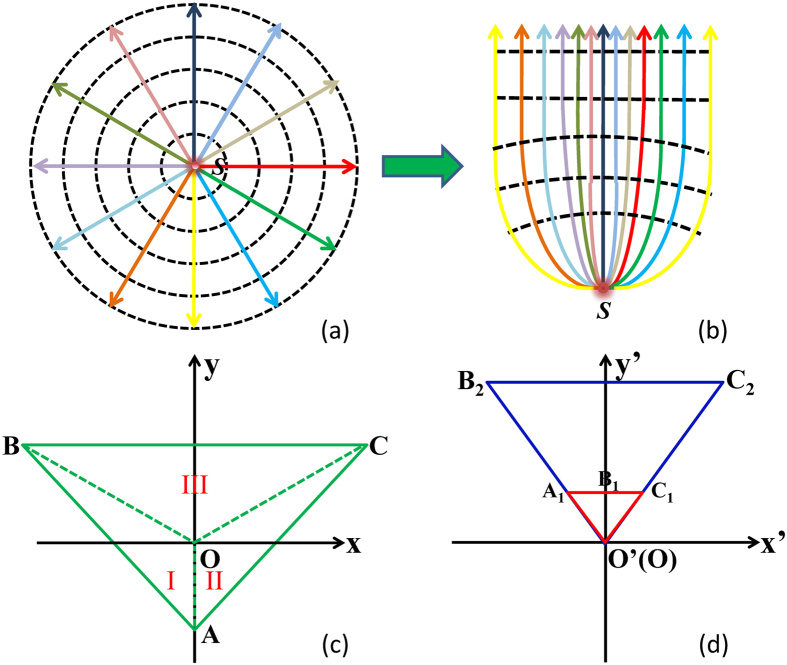
The schematic of the one-way behavior of electromagnetic wave. The power flow (solid line) and wavefront (dash line) of a point source in free space (**a**) and transformation medium (**b**). (**c**,**d**) Are the corresponding transformation space.

Figure 1(a,b) show the schematic of power flow and wavefront of a point source propagating in free space and a designed transformation medium, respectively. In the free space, as shown in Fig. 1(a), the wavefront of the point source is a circle, and the excited point source radiates into all directions. However, when the point source is located in the transformation medium (Fig. 1(b)), a Gaussian beam-like wavefront is produced, and the excited point source propagates only along one designated direction, resulting in the unidirectional emission. In order to achieve such a unidirectional behavior, we establish the mapping between Fig. 1(c,d) based on the coordinate transformation. Here, (x, y, z) and (x′, y′, z′) are coordinates in real space and virtual space, respectively. The regions of OAB and OAC in real space are bended and compressed into the regions of O’A_1_B_1_ and O’C_1_B_1_ in virtual space, respectively. Meanwhile, the region OBC in real space is stretched into the region O’B_2_C_2_ in virtual space. According to the transformation optics, the coordinate transformation between Fig. 1(c,d) can be expressed as follows:

In region I:


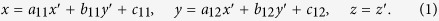


In region II:


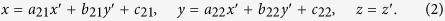


In region III:


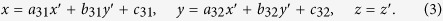


where,


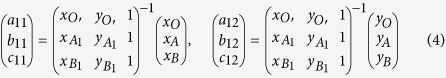



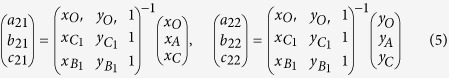



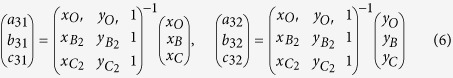


The corresponding transformation medium parameters from Eqs (1,2,3) can be expressed:

In region I:


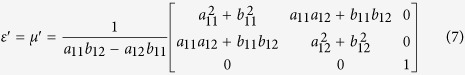


In region II:


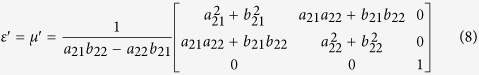


In region III:


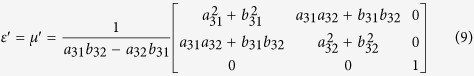


It should be noted that the transformation medium in region I, II, and III are anisotropic and homogenous, so it can hardly realize by naturally occurring crystals. It can be realized by using traditional metamaterial technology (such as the SRR (split resonance rings)) and effective theory (i.e., to further reduce transformation medium parameters), but limited to the resonance bandwidth. Although, the GRIN metamaterial technology (see ref. 13) can also be applied to realize the same phenomenon with a broad bandwidth, it cannot be simpler than our method of the L-C circuits.

## Results and Discussions

**Figure 2:**
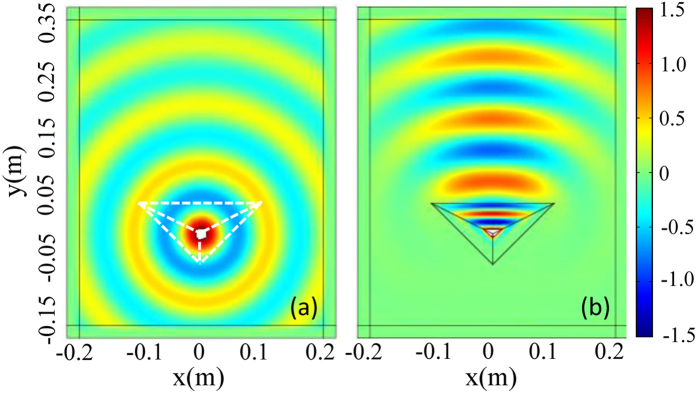
The electric field distribution of a point source embedded in free space (**a**) and transformation medium (**b**), respectively. The corresponding coordinates in this case are: O’(0, 0), A(0, −0.05 m), B(−0.1 m, 0.05 m), C(0.1 m, 0.05 m), O(0, 0), A_1_(−0.01 m, 0.02 m), B_1_(0, 0.02 m); C_1_(0.01 m, 0.02 m), B_2_(−0.1 m, 0.2 m), C_2_(0.1 m, 0.2 m).

The numerical simulations of unidirectional effects were performed using COMSOL Multiphysics^®^. Figure 2(a), shows the electric field distribution of a point source located at (0, 0) with frequency of 3 GHz in free space. The wavefront of the electric field is a circle, which means that the field is radiated isotropically. However, when the same point source is embedded in a properly designed transformation medium (the corresponding parameters is shown in Eq. (7)~Eq. (9)), the radiated field is mostly directed upward as shown in Fig. 2(b), validating the unidirectional emission of the electromagnetic waves. In this case, the wave excited by the point source only propagates in the +y direction. We want to emphasis that the similar phenomenon can also be realized when the excitation source is located at other positions such as (0, 0.015 m) and (0, −0.01 m) (see Supplementary Fig. S1), indicating that the position of excitation source is no longer fixed at (0, 0) but flexible.

**Figure 3:**
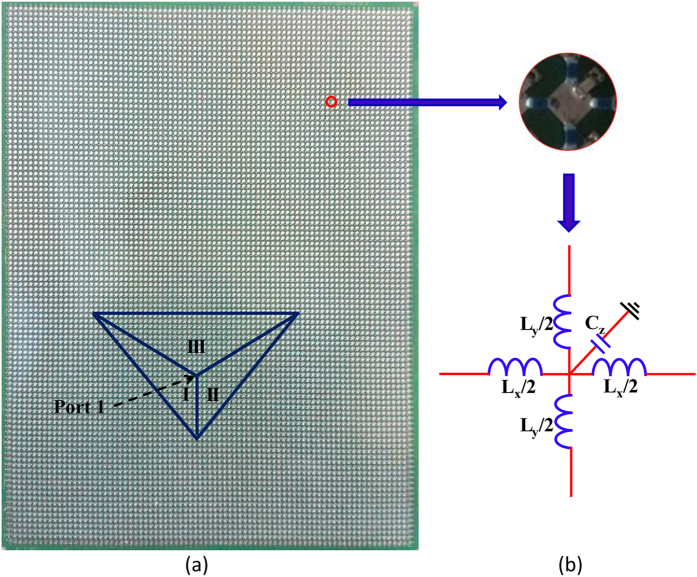
(**a**) An experimental device with transformation medium in the triangle region. (**b**) Unit cell of the L-C network.

Experimentally, such unidirectional behavior effects based on transformation optics can be realized by using a network of properly designed and implemented L-C transmission lines. A fabricated periodic L-C transmission line network shown in Fig. 3(a) acts as the desired transformation medium (region I, II, and III) and the background (the space outside the region I, II, and III). Figure 3(b) depicts the unit cell of L-C transmission line network. If *L*_*x*_ is equal to *L*_*y*_, the L-C transmission line network can be regarded as an isotropic medium with positive permeability and permittivity due to the mapping between the L-C network equations and Maxwell’s equations. Therefore, the transformation medium can be precisely designed by selecting proper parameters of inductors and capacitors. When the long wavelength limit is considered, *i.e,* the model of effective medium, where the dimension (period) of the unit cell is much smaller than the wavelength of the excitation source, the relationship between the real material parameters and L-C transmission line network can be described as follows:


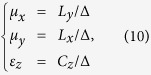


where Δ = 5mm is the length of the unit cell (here, the period Δ = 5mm is much smaller than the wavelength of the excited source). Like in refs 39 and 40, (here, in order to simplify the fabrication, the off-diagonal components of the transformation medium (in Eq. (7)~Eq. (8)) are also ignored like in refs 39 and 40, and such an approximation can also ensure that the unidirectional effect is verified in present experiment (see the measured results).) the detailed parameters can be chosen as listed in Table 1. The fabrication and measurement of the device will be discussed in details in Methods.

**Figure 4:**
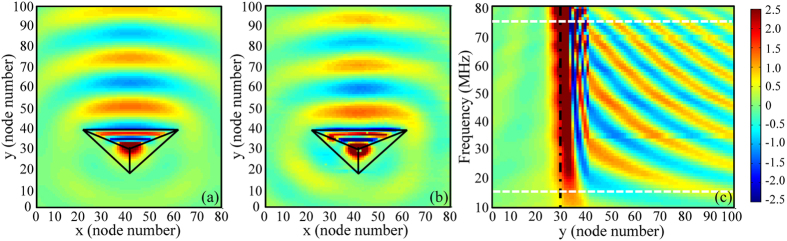
Simulated (**a**) and measured (**b**) node voltage distribution of the one-way behavior of electromagnetic waves at 45 MHz. (**c**) The simulated node voltage distribution at x = 41 (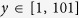), with excited frequency ranging from 10 MHz to 80 MHz.

The unidirectionality for this special medium was studied by both numerical simulations and experimental verifications, as shown in Fig. 4. Figure 4(a1) depicts the simulated voltage distribution of a point source (with frequency of 45 MHz) excited at the node (31, 41), based on the Agilent’s advanced design system (ADS). In this configuration, the excited source is coated with the transformation medium. So, almost all of the radiated waves (voltage distribution) from the point source propagate along the +y direction, while the radiation in other directions is significantly suppressed, indicating the unidirectional/directive emission of waves. Meanwhile, we fabricate the corresponding sample (Fig. 3(a)) with 81 grid nodes along x direction and 101 grid nodes along y direction to demonstrate this phenomenon. Figure 4(b) illustrates the measured voltage distribution of the excited point source located at the node (41, 31) with the frequency of 45 MHz. The excited point source can also just radiate into the +y direction, resulting in unidirectional/directive emission of electromagnetic waves. In comparison with Fig. 4(a,b), both of them have nearly the identical voltage distributions, which demonstrate that the numerical simulation and experimental measurement are matched with each other. In addition, Fig. 4(c) shows voltage distributions of our proposed device at *x* = 41 (Here, 41 is the node number, and 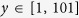) with various excited frequencies. From 15 MHz to 75 MHz, the intensity of voltage distribution at y > 30 is much stronger than that of y < 30, indicating that the designed unidirectional device can be worked in a broadband region (about from 15 MHz to 75 MHz).

**Figure 5:**
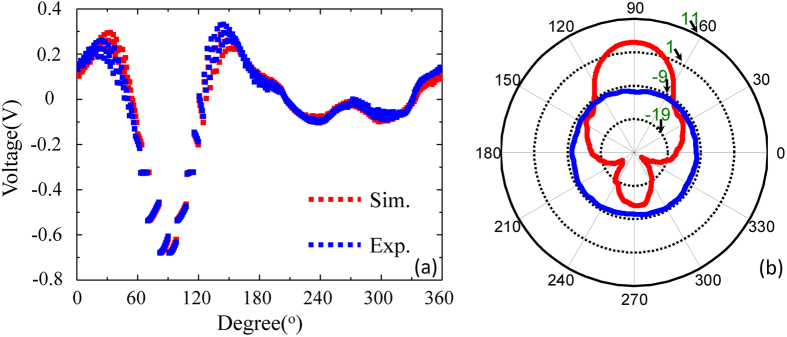
(**a**) Simulated (red curve) and measured (blue curve) voltage distributions of the device at r = 200 mm. (**b**) Simulated far-field patterns of the proposed device with (red curve) and without (blue curve) the transformation medium.

Now, we discuss the directivity of our designed unidirectional device, as shown in Fig. 5. Figure 5(a) shows the simulated and measured voltage distributions of our designed device at *f* = 45 MHz. The calculated voltage distributions show agreement with the measured results except for a slight difference because of the non-ideal electric capacitance and electric inductance. Figure 5(b) plots the simulated far-field patterns of the point source in the background medium (blue curve) and the transformation medium (red curve), respectively. The antenna directivity at *f* = 45 MHz is 14.3 dB. When compared with traditional lens directive antennas (such as a broadband half Maxwell fish-eye lens antenna in ref. 13), the designed device in this paper also presents excellent directivity. In addition, the far-field patterns for the designed device without/with reflectors and just with the bare reflector (without the transformation medium) are shown in the supplementary of Fig. S2.

**Figure 6:**
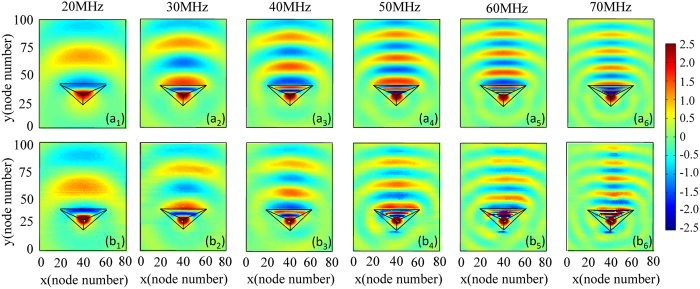
The node voltage distribution of the device with the corresponding excited frequency of 20, 30, 40, 50, 60, 70 MHz. The first and the second line are corresponding to the simulated and the measured results, respectively.

Next, we illustrate the voltage distributions of all nodes in the transmission line network to further verify the broadband characteristic of unidirectionality. Figure 6(a1,a6) depict the corresponding voltage distributions of device at the design frequencies of 20, 30, 40, 50, 60 and 70 MHz. For different excitation frequencies, our designed device also shows the unidirectional property with antenna directivity of 12.5, 13.5, 13.9, 14.65, 16.85 and 16.95 dB, respectively. Figure 6(b1,b6) present the measured results of such sample at the frequencies of 20, 30, 40, 50, 60 and 70 MHz, which are in agreement with the simulation results, demonstrating that our designed sample can work in a broad frequency range.

Finally, we give a brief discussion on the limitations of the designed device. Although the proposed device shows broadband of unidirectional emitting (which is originated from the non-resonant nature of the transmission line metamaterials), it has more or less limitations. First, the size of the proposed directional emitting device is still much bigger, which is limited to be applied in the SOC (system of chip). Second, such a conceptual verification of broadband unidirectional emitting can be just realized in RF or microwave region. It can also be realized in THz and visible frequency based on the metamaterial technology such as the SRR, it is limited to the bandwidth due to the resonance property. Therefore, to realize the broadband unidirectional emitting based on TO in high frequency region, is still an urgent problem to be solved. But, we still want to emphasis that it is a new or another method to design broadband directive antennas. On the other hand, if the design is extended to a three-dimensional (3D) case, the permittivity and permeability in Eqs 7~9 are further complicated. Actually, in the 3D case, the corresponding coordinate transformations are between (x, y, z) and (x′, y′, z′), and each component in permittivity and permeability tensors (Eqs 7~9) is non-zero, which means that the original parameters (in two-dimensional case) are not feasible. In a word, such a TO-based method can be extended into 3D cases, but the parameters are much more complicated, resulting in the limitation in fabrication.

In conclusion, we have proposed a new method to generate unidirectional emitting of electromagnetic waves based on transformation optics. The transformation medium mimicked by L-C transmission line networks was designed to realize the unidirectionality of electromagnetic waves. In particular, it is also demonstrated that such a device can work in a broadband frequency region (from 15 MHz to 75 MHz). Therefore, such a conceptual verification of unidirectional device may pave the way to realize the TO-based high-directive antennas.

## Methods

### Sample fabrication

The device is fabricated on a grounded flame retardant 4 (FR4) substrate with 1 mm-thickness and the dielectric constant of *ε*_*r*_ = 4.3. There are 81 grid nodes (along x direction) and 101 grid nodes (along y direction) on the substrate. The distance between two adjacent nodes is 5 mm. 0603 package size of the surface-mounted capacitors and inductors are applied to simulate the transformation medium and background. Each unit cell of the L-C transmission line networks is consisted of four surface-mounted inductors in series and one capacitor in shunt to the ground by a via-hole. The boundary of the device is truncated by using the Bloch impedances. The whole size of our structure is about 430 mm × 530 mm.

### Experimental measurement

Agilent E5071C VNA is applied to measure the device. Port 1 of the VNA is connected to node (31, 41) to provide the excitation source, while port 2 of the VNA connected to a high-impedance amplifier can scan over the sample surface to gather the transmission coefficient of each node. And, the voltage and electric field of nodes can be extracted from the transmission coefficients.

## Additional Information

**How to cite this article:** Zang, X. F. *et al*. Broadband unidirectional behavior of electromagnetic waves based on transformation optics. *Sci. Rep.* **7**, 40941; doi: 10.1038/srep40941 (2017).

**Publisher's note:** Springer Nature remains neutral with regard to jurisdictional claims in published maps and institutional affiliations.

## Supplementary Material

Supplementary Information

## Figures and Tables

**Table 1:** The unit cell parameters of the sample and the relative permittivity and permeability.

Vaule	Region		
	Background	Region I/II	Region III
L_x_(nH)	18	75	4.3
L_y_(nH)	18	91	75
C_z_(pF)	51	2.2	220
μ_x_	1	4.17	4.16
μ_y_	1	5.05	0.24
ε_z_	1	0.043	4.3

Background refers to the medium outside the region I, II, and III.
