# Supplementary material for: Broadband unidirectional behavior of electromagnetic waves based on transformation optics
Source: Sci Rep. 2017 Jan 20;7:40941. doi: 10.1038/srep40941 (PMC5247730; doi:10.1038/srep40941)
Supplement: Supplementary Information [file srep40941-s1.doc]

**Broadband unidirectional behavior of electromagnetic waves based on transformation optics**

**XiaoFei Zang^1,2^, YiMing Zhu^1,2,*^, XueBin Ji^1^, Lin Chen^1,2^, Qing Hu^3,1^, and SongLin Zhuang^1,2^**

1. *Shanghai Key Lab of Modern Optical System and Engineering Research Center of Optical Instrument and System, Ministry of Education,, University of Shanghai for Science and Technology, No.516 JunGong Road, Shanghai 200093, P. R. China.*
2. *Terahertz Science Cooperative Innovation Center, Chengdu 610054, P. R. China.*
3. *Department of Electrical Engineering and Computer Science and Research Laboratory of Electronics, Massachusetts Institute of Technology, 77 Massachusetts Avenue. Cambridge, Massachusetts 02139, USA*

Figure S1 Electric field distributions of a point source embedded at (0, 0.015m) (a) and (0, -0.01m) (b).

Figures S1 (a) and S1 (b) show that the unidirectional emission can be realized with the excited source located at (0, 0.015*m*)) and (0, -0.01*m*)). In a word, the excited point source is not limited to locate at (0, 0) (it doesn’t like the case of dielectric singularities). Therefore, we think that the position of the excited point (line) source is flexible rather than fixed.

Figure S2 Simulated far-field patterns: blue and red curves for the designed device without and with reflector, respectively; black curve for the case of just with the bare reflector (without the transformation medium).

Figure S2 illustrates the far-field patterns for the designed device without/with reflectors and just with the bare reflector (without the transformation medium), respectively. When we add a corner reflector in the edge of the transformation medium, the far-field profile is nearly the same as the case of without the reflector, as shown in Fig. S2 (the red and blue curves). The corresponding directivity is 14.45 dB and 14.3 dB, respectively. In fact, a corner reflector (with the transformation medium embedded in the corner reflector) can be applied to achieve nearly the same results of the directivity (although the back radiation (for example at θ=270^0^) is better than that case of without the reflector). However, for the case of just a bare corner reflector (without the transformation medium embedded in the corner reflector), the corresponding directivity is 9.4 dB, which is smaller than our designed device.
